# Estimated Costs of False Laboratory Diagnoses of Tuberculosis in Three Patients

**DOI:** 10.3201/eid0811020387

**Published:** 2002-11

**Authors:** Jill M. Northrup, Ann C. Miller, Edward Nardell, Sharon Sharnprapai, Sue Etkind, Jeffrey Driscoll, Michael McGarry, Harry W. Taber, Paul Elvin, Noreen L. Qualls, Christopher R. Braden

**Affiliations:** * Massachusetts Department of Public Health, Boston, Massachusetts, USA; † Harvard Medical School, Boston, Massachusetts, USA; ‡ New York State Department of Health, Wadsworth Center, Albany, New York, USA; § Centers for Disease Control and Prevention, Atlanta, Georgia, USA

**Keywords:** Costs and cost analysis, DNA fingerprinting, *Mycobacterium tuberculosis*, restriction fragment length polymorphism, tuberculosis

## Abstract

We estimated direct medical and nonmedical costs associated with a false diagnosis of tuberculosis (TB) caused by laboratory cross-contamination of Mycobacterium tuberculosis cultures in Massachusetts in 1998 and 1999. For three patients who received misdiagnoses of active TB disease on the basis of laboratory cross-contamination, the costs totaled U.S.$32,618. Of the total, 97% was attributed to the public sector (local and state health departments, public health hospital and laboratory, and county and state correctional facilities); 3% to the private sector (physicians, hospitals, and laboratories); and <1% to the patient. Hospitalizations and inpatient tests, procedures, and TB medications accounted for 69% of costs, and outpatient TB medications accounted for 18%. The average cost per patient was $10,873 (range, $1,033-$21,306). Reducing laboratory cross-contamination and quickly identifying patients with cross-contaminated cultures can prevent unnecessary and potentially dangerous treatment regimens and anguish for the patient and financial burden to the health-care system.

To date, studies investigating cases of laboratory cross-contamination have described only the resources to care for patients who received false diagnoses of tuberculosis (TB) (1-6); to our knowledge, the costs attributable to cross-contamination have not been reported. We estimated direct medical and nonmedical costs for three patients whose misdiagnoses of active TB disease resulted from laboratory cross-contamination of Mycobacterium tuberculosis cultures. The costs totaled U.S.$32,618. By examining the costs from the perspective of the patient and the public and private sectors, we documented the financial costs to the health-care system caused by laboratory cross-contamination.

The rate of patients having false-positive M. tuberculosis cultures resulting from laboratory cross-contamination may be up to 33% of culture-confirmed TB patients (1-3,7-11). Reportedly two thirds of patients with false-positive cultures are treated for active TB disease (4) and may undergo unnecessary, potentially toxic anti-TB therapy. Public health departments may initiate costly activities such as contact investigations and directly observed therapy. Dunlap et al. report that if persons who receive misdiagnoses resulting from laboratory cross-contamination were treated as TB case-patients with contact investigations and 6 months of directly observed therapy, the costs to the health-care system would be $2,500 per patient (12) in 1993 U.S. dollars, or $3,111 in 1999 dollars, when the Medical Care component of the Consumer Price Index is used to convert 1993 dollars to 1999 dollars.

## Methods

### Identifying Patients

As part of the Centers for Disease Control and Prevention-funded National Tuberculosis Genotyping and Surveillance Network, the Massachusetts Department of Public Health, Division of Tuberculosis Prevention and Control (TB Division), conducted a population-based study to determine the rate of TB misdiagnosis in Massachusetts caused by laboratory cross-contamination of M. tuberculosis specimens.

The study also evaluated the following criteria that may assist TB control programs to identify patients with potentially cross-contaminated cultures: 1) the patient had a single respiratory specimen positive for M. tuberculosis, regardless of acid-fast bacilli (AFB) smear status; a single extrapulmonary body fluid specimen positive for M. tuberculosis, regardless of AFB status; or a single tissue specimen positive for M. tuberculosis without evidence of AFB or granuloma on histologic examination; 2) the patient had an M. tuberculosis culture-positive specimen collected >30 days after the collection of an M. tuberculosis culture-negative specimen, and the isolate had a unique genotype compared with any previous isolate from the same patient; 3) the patient had an M. tuberculosis culture-positive specimen collected >90 days after the start of appropriate, continuous anti-TB therapy, and the isolate had a unique genotype compared with any previous isolate from the same patient; 4) a caretaker indicated that an M. tuberculosis culture-positive result was clinically inconsistent; or 5) a laboratorian indicated that the M. tuberculosis culture-positive result might be false.

The Massachusetts Department of Public Health Human Research Review Committee reviewed the protocol and waived oversight. Personnel in 24 mycobacteriology laboratories (all the laboratories that were processing specimens for AFB for persons in Massachusetts at the time) and public health professionals worked together to identify patients with potentially cross-contaminated specimens. Persons who were reported in Massachusetts as possible TB patients and were reported as having M. tuberculosis-positive cultures between January 1, 1998, and June 30, 1999, were prospectively screened. Persons meeting one or more of the criteria were included in the study.

We reviewed laboratory records to identify potential sources of cross-contamination, i.e., any M. tuberculosis culture-positive specimen or laboratory control strain processed, reprocessed, or subcultured within 2 working days of the potentially cross-contaminated specimen. For laboratories that did not record usage dates for control strains, the controls were designated as potential sources of cross-contamination and were obtained for genotyping.

The genotype was determined for isolates by IS6110-based restriction fragment length polymorphism (RFLP) (13) at the Northeast Regional Genotyping Laboratory, Wadsworth Center, Albany, New York. Spoligotyping (14) was used as a secondary typing method for isolates with five or fewer IS6110 copies.

Patients with potentially cross-contaminated isolates that matched organisms from potential sources of contamination by genotype and patients for whom a DNA fingerprint could not be produced were investigated. Investigations included reviews of medical and public health department records, abstracts from laboratory data, and patient interviews. Because the criteria would potentially identify not only patients with false-positive M. tuberculosis cultures that resulted from laboratory cross-contamination but also patients with false-positive cultures that were caused by other errors as well as true TB cases, a panel of three TB investigators representing other sentinel sites in the genotyping network reviewed the findings. The panel judged whether laboratory cross-contamination was possible, likely, or unlikely and whether the patient had active TB disease or another clinical diagnosis.

### Estimating Costs

The cost of TB misdiagnosis was estimated retrospectively for patients who had M. tuberculosis culture-positive specimens judged to be possibly or likely caused by laboratory cross-contamination and who received inappropriate diagnoses and were treated for TB because of the false-positive results. (Patients judged to have false-positive M. tuberculosis cultures caused by other error were not included in the cost analysis.) Costs for the patient, public sector (local and state health departments, public health hospital and laboratory, and county and state correctional facilities), and private sector (private physicians, hospitals, and laboratories) incurred specifically as a result of the cross-contaminated cultures are included (Table 1). Cost information was collected from the time of initial misdiagnosis until the patient was no longer followed for active TB disease. If patients had other, unrelated medical costs at the same time, the TB medical officer (EN) determined which costs could be attributed to cross-contaminated cultures.

**Table 1:** Cost inventory for three patients who received misdiagnoses of active tuberculosis disease on the basis of laboratory cross-contamination of *Mycobacterium tuberculosis* specimens^a^

Patient	Public sector^b^	Private sector^c^
Direct medical costs		
TB medications	Outpatient visits TB medications and PPD DOT provision Tests and procedures Contact investigations Hospitalizations	Outpatient visits TB medications and PPD Tests and procedures Contact investigations Hospitalizations
Direct nonmedical costs		
	Case management^d^ Overhead^e^	

^a^TB, tuberculosis; PPD, purified protein derivative of tuberculin; DOT, directly observed therapy. ^b^Local and state public health departments, public health hospital and laboratory, and county and state correctional facilities. ^c^Private physicians, hospitals, and laboratories. ^d^Health department case management and administrative support. ^e^Overhead costs, including rent, utilities, and supplies.

Data were collected on direct medical and nonmedical costs for the following: public health department case management and administrative support; outpatient visits; TB medications (started, continued, or changed); directly observed therapy; tests and procedures (bacteriologic, radiologic, chemical, hematologic, pathologic, immunologic, bronchoscopic, and biopsy); health department and hospital contact investigations and diagnostic and treatment services for contacts; and hospitalizations or transfers to hospital isolation rooms. Indirect and intangible costs were excluded.

The resources expended for TB care and treatment were identified from records from these sources: local and state public health departments; inpatient and outpatient medical departments; hospital, clinic, and laboratory billing departments; pharmacies; and mycobacteriology laboratories. We obtained information about contact investigations from health department and hospital infection control personnel, and we asked nurses about public health case management.

Cost estimates were obtained from several sources described below; detailed cost information for these estimates are available upon request. Public health department personnel costs for case management, administrative support, directly observed therapy provision, and contact investigations were estimated by multiplying the sum of annual salaries, fringe benefits, and overhead (rent, utilities, and supplies) by the fraction of the year spent on the activity (as estimated by the health department staff). Costs for providing directly observed therapy at a correctional facility were estimated by multiplying hourly salary by the number of hours spent on the activity, as estimated by the health services administrator. Costs for outpatient visits to health department TB clinics and for tests and procedures at these clinics were based on the TB Division's reimbursement to the clinics. Costs of private outpatient visits, tests, and procedures were estimated on the basis of information from provider and laboratory billing departments. Costs of TB medications and purified protein derivative (PPD) of tuberculin were based on the TB Division's expenditures for TB drugs and PPD for state fiscal years 1999-2000. The mycobacteriology supervisors estimated costs for mycobacteriology procedures at the public health laboratory and one private laboratory. Charges for hospitalizations and inpatient tests and procedures were obtained from patient billing records and were adjusted to market prices by using Medicare provider-specific, cost-to-charge ratios. The medical services' senior financial analyst estimated the costs for hospitalization at a correctional facility's infirmary on the basis of a flat daily bed rate.

### Calculating Cost

Total costs by health-care sector, cost category, and patient were calculated and reported in 1999 U.S. dollars and rounded to the nearest whole dollar. 1998 dollars were adjusted by using the Consumer Price Index Medical Care component. Costs were not discounted because all costs occurred within 1 year of diagnosis.

## Results

### Rate of TB Misdiagnosis

Between January 1, 1998, and June 30, 1999, 342 of the persons reported as possible TB case-patients in Massachusetts had M. tuberculosis positive cultures; of these, 5 (1.5%) had cultures judged to be cross-contaminated in the laboratory. Three (0.9% of 342) of the five persons received misdiagnoses for active TB disease on the basis of the results (Table 2). Each case had been reported as a verified case of TB for national surveillance, but the status was revoked when information from this investigation became available.

**Table 2:** Characteristics of patients who received misdiagnoses of active tuberculosis disease resulting from laboratory cross-contamination of *Mycobacterium tuberculosis* specimens^a^

Characteristics	Patient 1	Patient 2	Patient 3
Demographic information			
Age at diagnosis (yrs)	59	29	38
Sex	Female	Male	Male
Clinical information			
Site of disease	Lymphatic	Pulmonary	Soft tissue, right index finger
Symptoms when examined	Chronic cough, weight loss, increasing fatigue, night sweats (Sept 1998)	Abdominal discomfort, diarrhea, flank pain, high fever, cough with blood, delirium tremens (Nov 1998)	Infection of right index finger,^b^ great pain, lymphangitic streaks up arm (Aug 1998)
Radiology, initial	CAT scan: lymphadenopathy, densities in upper lobes suggestive of infiltration or scarring	Chest x-ray: right lower lobe infiltrate, improved with intravenous ceftriaxone	X-ray right hand: swelling over right index DIP and PIP joints; chest x-ray: normal
Pathology	Lymph node biopsy positive for lymphoma, chemotherapy started	Not applicable	Not done/missing
TST result	Negative	Negative	Negative
Underlying conditions and TB risk factors	History of Hodgkin lymphoma and treatment for active TB disease in 1995,^c^ non–U.S.-born	History of chronic alcohol abuse and cocaine use	HIV positive, history of IVDU and incarceration
TB health care			
TB health-care provider	Private physician	Public health department TB clinic	Public health department TB clinic, correctional facility clinic
Type of TB therapy	Self-administered	Daily DOT by public health nurse	Daily DOT by correctional facility staff
Duration of TB therapy	<1 month (started Dec 1998)	<2 months (started Dec 1998)	11 months (treated for 2 weeks in Oct 1998, restarted December 1998)
Hospitalization(s) following TB diagnosis	5 days in private hospital (Jan 1999) with increasing respiratory distress, treated for community acquired pneumonia, died of presumed progression of non–Hodgkin lymphoma	11 days in private hospital with acute gastritis secondary to alcohol abuse (Jan 1999), TB therapy discontinued secondary to increased LFTs; 15 days at public health hospital for TB management; TB ruled out	8 days at public health hospital to start anti-TB therapy and rule out pulmonary and bone involvement (Oct 1998); 5 days in correctional facility infirmary
Contact investigations			
By public health department	Not done	One household contact identified, TST-negative	Not done
By hospital infection control	Not done	Not done	Not done
Information on cross-contaminated specimen			
Specimen type	Right inguinal lymph node tissue	Sputum	Swab of finger cellulitis
AFB smear result	Negative	Negative	Negative
AFB culture result	1 colony at 60 days (reported Dec 1998), sensitive to INH, RIF, EMB, Strep (PZA not tested)	1 colony at 40 days (reported Dec 1998), slightly resistant to INH	“Rare” colonies at 42 days (reported Sept 1998), INH resistant
NTGSN IS*6110* RFLP analysis	10-band pattern (reported April 1999), RFLP match to an isolate from a known TB patient	9-band pattern (reported April 1999), RFLP match to an isolate from a known TB patient	16-band pattern (reported Oct 1999), RFLP match to laboratory control strain H37Ra
Case appraisal results^d^			
Case diagnosis	Lymphoma, nosocomial bacterial pneumonia	Community-acquired pneumonia	Streptococcus cellulitis
Did laboratory cross-contamination occur?	Likely	Likely	Likely

^a^TST, tuberculin skin test; TB, tuberculosis; CAT, computerized axial tomograpy; AFB, acid-fast bacilli; NTGSN, National Tuberculosis Genotyping and Surveillance Network; RFLP, restriction fragment length polymorphism; INH, isoniazid; RIF, rifampin; EMB, ethambutol; Strep, streptomycin; PZA, pyrazinamide; DOT, directly observed therapy; LFTs, liver function tests; DIP, distal interphalangeal; PIP, proximal interphalangeal; and IVDU, intravenous drug use. ^b^Infection of right index finger ultimately resulting in amputation; specimen grew Streptococcus Group A. ^c^Patient treated for active TB disease in 1995, although there was not enough evidence to verify the case for national surveillance. ^d^Case appraisals performed by a panel of three TB investigators representing other NTGSN sentinel sites.

Despite their positive cultures, two patients with cross-contaminated cultures were not treated for active TB disease, largely because their physicians did not believe a TB diagnosis was clinically consistent. The mycobacteriology laboratory that processed one patient's specimen questioned the result and performed in-house RFLP typing that confirmed laboratory cross-contamination. Both patients were informed about the false-positive results and reassured about the findings.

### Costs by Health-Care Sector

The costs of caring for the three patients whose misdiagnoses and treatment for active TB resulted from laboratory cross-contamination are summarized in Table 3. The total was estimated to be $32,618 in 1999 U.S. dollars. Ninety-seven percent of costs ($31,552) occurred within the public sector: $14,319 at the public hospital, $9,024 within the correctional system, $7,075 to local and state public health departments, and $1,134 to the public health laboratory. Three percent ($949) occurred within the private sector: $381 at hospitals, $316 from laboratories, and $252 for physicians. The patient incurred <1% of the total costs-$118 that went for TB medications.

**Table 3:** Estimated costs for three patients who received misdiagnoses of active tuberculosis disease on the basis of laboratory cross-contamination of *Mycobacterium tuberculosis* specimens^a,b^

Cost category	Estimated costs (U.S.$)			
	Patient 1	Patient 2	Patient 3	Total
Case management^c^	226	288	100	614
Outpatient visits	186	58	443	687
TB medications and PPD	175	606	5,061	5,842
DOT provision^d^	0	508	868	1,376
Tests and procedures	134	1,904	2,703	4,741
Contact investigations^e^	0	10	0	10
Hospitalizations^f^	312	6,905	12,131	19,348
Total	1,033	10,279	21,306	32,618

^a^Costs reported in 1999 U.S. dollars, rounded to the nearest whole dollar. Costs adjusted to 1999 dollars by using the Medical Care group of the Consumer Price Index. ^b^TB, tuberculosis; PPD, purified protein derivative of tuberculin; DOT, directly observed therapy. ^c^Personnel time for health department case management and administrative support. ^d^Personnel time to provide directly observed therapy ^e^Personnel time to perform contact testing. ^f^Daily inpatient bed rate and differential for transfer to isolation room.

### Costs by Category

Across all sectors, hospitalizations (daily inpatient bed rate and differential for transfer to isolation room) accounted for 59% ($19,348) of total costs. This category was followed by TB medications and PPD ($68 inpatient/$5,774 outpatient), tests and procedures ($3,046 inpatient/$1,695 outpatient), personnel time for directly observed therapy provision ($1,376), outpatient visits ($686), personnel time for health department case management and administrative support ($615), and personnel time for contact investigations ($10). In all, $22, 462 (69%) of the total cost came from hospitalizations and inpatient TB medications, tests, and procedures.

### Costs by Patient

The total costs for health care for patients 1, 2, and 3 were $1,033, $10,279, and $21,306, respectively. The average cost per patient was $10,873. Sixty-seven percent of the costs for patient 1 occurred in the private sector: $369 at the hospital, $253 for physicians, and $67 from the laboratory; 22% ($226) by public health departments for case management and administrative support; and 11% ($118) by the patient for TB medications. For patient 2's care, 97% of the costs occurred in the public sector: $7,809 at the public health hospital, $1,491 to health departments, and $720 to the public health laboratory. Three percent of the costs occurred in the private sector: $248 from the laboratory and $11 at the hospital. All costs for patient 3 were within the public sector with $9,024 to county and state correctional facilities, $6,510 at the public health hospital, $5,358 to public health departments, and $414 to the public health laboratory.

## Discussion

### Rate of TB Misdiagnosis

In Massachusetts, the rate of patients having false-positive cultures resulting from laboratory cross-contamination of M. tuberculosis specimens was 1.5% of the culture-confirmed possible TB cases. This rate is within the range demonstrated in other population-based studies (1-3,7-11). In our study, 60% of the patients with cross-contaminated cultures received misdiagnoses and were treated for active TB disease, yielding a rate of TB misdiagnoses caused by laboratory cross-contamination of 0.9% of patients with culture-confirmed TB. These findings corroborate those of Burman and Reves, who estimated that two thirds of patients with false-positive cultures are treated for active TB (4).

### Costs of Misdiagnosis

For the three patients, the costs of TB false diagnoses from laboratory cross-contamination fell largely to the public health and correctional system. Hospitalizations and inpatient tests, procedures, and TB medications accounted for 69% of total health-care sector costs. This finding is consistent with the findings of Brown et al., who demonstrated that inpatient care accounted for 60% of TB health-care expenditures in 1991 even though TB is considered an ambulatory disease (15). Moreover, most of the inpatient costs were for the care of patients 2 and 3, whose underlying circumstances contributed to their hospitalization.

The costs of TB misdiagnosis varied greatly between the three patients (range $1,033-$21,306) and reflected their unique clinical circumstances and treatment courses. Patient 1 had the lowest costs of the three patients. She was treated with anti-TB therapy for only a few weeks before she died of probable lymphoma. Because extrapulmonary TB was diagnosed, no contact investigation was conducted.

Patient 2, with cost of care totaling $10,279, completed <2 months of anti-TB therapy. It was discontinued secondary to elevated liver function tests. The physician found no clinical correlation for a TB diagnosis and did not restart treatment, thus averting additional costs for a full 6-month course of TB therapy. Although pulmonary TB was diagnosed, the patient was AFB smear-negative, so only a limited contact investigation was performed: one household contact was tested.

The greatest cost ($21,306) was for patient 3. The patient's false-positive culture was discovered only as a result of genotyping through the TB genotyping network, and the patient completed 11 months of an intended 12-month course of anti-TB therapy. A diagnosis of single drug-resistant TB and an HIV-positive status further complicated his care. Because extrapulmonary TB was diagnosed, no contact investigation was conducted.

The average cost per patient was $10,873; however, because of the small sample size (n=3), we cannot conclude whether this is a representative estimate of the average cost per TB misdiagnosis. Since two of the patients were hospitalized in a public, long-term care facility rather than an acute-care hospital, the costs were probably much lower than they could have been. However, these preliminary findings demonstrate that substantial costs can result from misdiagnoses caused by laboratory cross-contamination. Additional research with a larger sample size is warranted.

### Limitations to the Study

This study included only three patients and did not include estimates of indirect and intangible costs. Since these costs largely affect the patient, we likely underestimated the effects of TB misdiagnosis on patients. Although the consequences were not collected formally, the patients had negative indirect or intangible consequences attributable to the misdiagnoses. The hospitalization of patient 2 and patient 3 represented 13 and 15 days of lost productivity, respectively. In addition, patient 3 underwent a painful bone marrow biopsy to rule out TB involvement of the bone. At the county correctional facility, patient 3 was placed in solitary confinement after testing positive for an illegal substance because of a false-positive reaction from rifampin. The patients likely experienced emotional anxiety, fear, stress, and stigmatization; they were also exposed to unnecessary treatment with potential risks for adverse effects (which did not occur).

Another limitation is that personnel time costs were derived from staff's retrospective estimates of the time involved in various activities, which could have resulted in error. We excluded payers such as Medicare, Medicaid, or private insurers from our study. We showed where costs were incurred within the health-care sectors, but we did not address who actually paid for the resources. This is another area for future research.

### Averting Costs of TB Misdiagnoses

This study demonstrates that substantial financial burden can be placed on the health-care system as a result of laboratory cross-contamination. The study also underscores the need for primary prevention of laboratory cross-contamination and the timely recognition of patients who have cross-contaminated M. tuberculosis cultures.

Investigators have recommended actions that laboratories, clinicians, and health departments can take to minimize the negative consequences of false-positive M. tuberculosis cultures: standardizing laboratory procedures, establishing surveillance for identifying false-positive M. tuberculosis cultures, and prospectively screening for patients who may have false-positive cultures (1,4-6,9). We found that clinicians may play an important role in averting the costs associated with TB misdiagnosis resulting from laboratory cross-contamination. Of the five patients with cross-contaminated M. tuberculosis cultures, two were not diagnosed with TB because their physicians did not believe the false-positive results. Anti-TB therapy was discontinued after only 2 months for patient 2 because his TB care provider did not believe a TB diagnosis was clinically consistent. Thus interventions may be targeted at physicians who submit samples that test positive for M. tuberculosis.

Selective genotyping of isolates from patients who have single positive M. tuberculosis cultures may also play a role in limiting the costs of TB misdiagnosis resulting from laboratory cross-contamination. For two of the three study patients who received misdiagnoses for active TB disease, neither the clinicians nor the laboratory personnel reported having suspected that the M. tuberculosis culture-positive result might be false; the errors were only detected through routine genotyping by the TB genotyping network.

Actions taken to minimize the negative consequences of false-positive M. tuberculosis cultures would require health-care resources. Even so, the costs of implementing these actions would likely be less than the costs of misdiagnosis, especially when intangible costs to the patient are considered. Increased efforts to avoid laboratory cross-contamination and to detect its occurrence as quickly as possible could help prevent unnecessary and potentially dangerous treatment, anguish for the patient, and financial costs to the health-care system.
